# Tumor Necrosis Factor Alpha rs1800629 Polymorphism and Risk of Cervical Lesions: A Meta-Analysis

**DOI:** 10.1371/journal.pone.0069201

**Published:** 2013-08-27

**Authors:** Min Li, Ying Han, Ting-Ting Wu, Yichen Feng, Hong-Bo Wang

**Affiliations:** 1 Department of Obstetrics and Gynecology, Union Hospital, Tongji Medical College, Huazhong University of Science and Technology, Wuhan, China; 2 Department of Obstetrics and Gynecology, South Branch of the Six People's Hospital Affiliated to Shanghai Jiao Tong University, Shanghai, China; IPO, Inst Port Oncology, Portugal

## Abstract

**Background:**

Tumor necrosis factor- alpha (TNF-α) is an inflammatory cytokine which may play important role on the immune response may control the progression of cervical lesions. There is a possible association between TNF-α rs1800629 G/A polymorphism and cervical lesions, but previous studies report conflicting results. We performed a meta-analysis to comprehensively assess the association between TNF-α rs1800629 polymorphism and cervical lesions risk.

**Methods:**

Literature searches of Pubmed, Embase, Web of Science, and Wanfang databases were performed for all publications on the association between TNF-α rs1800629 polymorphism and cervical lesions through December 15, 2012. The pooled odds ratios (ORs) with their 95% confidence interval (95%CIs) were calculated to assess the strength of the association.

**Results:**

Twenty individual case-control studies from 19 publications with a total of 4,146 cases and 4,731 controls were finally included into the meta-analysis. Overall, TNF-α rs1800629 polymorphism was significantly associated with increased risk of cervical lesions under two main genetic comparison models (For A versus G: OR 1.22, 95%CI 1.04–1.44, P = 0.017; for AA versus GG: OR 1.32, 95%CI 1.02–1.71, P = 0.034). Subgroup analysis by ethnicity further showed that there was a significant association between TNF-α rs1800629 polymorphism and increased risk of cervical lesions in Caucasians but not in Asians. Subgroup analysis by the types of cervical lesions showed that there was a significant association between TNF-α rs1800629 polymorphism and increased risk of cervical cancer (For A versus G: OR 1.24, 95%CI 1.05–1.47, P = 0.011; for AA versus GG: OR 1.31, 95%CI 1.01–1.70, P = 0.043; for AA/GA versus GG: OR 1.25, 95%CI 1.01–1.54, P = 0.039).

**Conclusion:**

The meta-analysis suggests that TNF-α rs1800629 polymorphism is associated with increased risk of cervical lesions, especially in Caucasians.

## Introduction

Cervical cancer is the third most common cancer and the fourth leading cause of cancer death among females, accounting for nearly 10% of the total newly-diagnosed cancer cases and 8% of the total cancer deaths [1]. Global incidence of cervical cancer has increased from about 378,000 cases per year in 1980 to about 454,000 cases per year in 2010 [2]. Risk factors of cervical cancer include human papillomavirus (HPV) and smoking, and infection of carcinogenic HPV is a main cause of almost all cases with cervical lesions [3]. Preinvasive lesions of cervical cancer have also been classified in terms of squamous intraepithelial lesions (SILs). Cervical cancer is considered to be a preventable disease because of its relatively long period of precancerous lesions, such as cervical intraepithelial neoplasia (CIN) [4]. Several cytokines that modulate the immunologic response have been implicated in the development of cervical cancer [5]–[7]. It has been well accepted that cervical cancer is mainly initiated by HPV infection, and tumor necrosis factor-alpha (TNF-α) is an inflammatory cytokine which may play important roles in the immune response of cervical lesions [5], [7]. TNF-α is located on chromosome 6 (region p21.3), and it is a potent proinflammatory cytokine playing an important role in the development of the immune response [5], [6]. Currently, there are several common single nucleotide polymorphisms (SNP) in the TNF-α gene which can regulate the transcription and production of TNF-α, such as TNF-α rs1800629 and TNF-α rs361525 [8], [9]. TNF-α rs1800629 is the most studied polymorphism, which is a G to A transition in the promoter at position −308 and is associated with the level of TNF-α expression [10], [11]. Therefore, TNF-α rs1800629 polymorphism may be related with risk of cervical lesions [10], [11]. However, results from previous studies examining the association between TNF-α rs1800629 polymorphism and risk of cervical lesions were contradictory [12]–[25]. These inconclusive results may due to the limited sample size of single study or the different characteristics among studies, such as ethnicity, pathological types, and sources of controls. Therefore, we performed a meta-analysis to comprehensively assess the association between TNF-α rs1800629 polymorphism and cervical lesions risk.

## Methods

### Search strategy

An electronic search of the Pubmed, Embase, Web of Science, and Wanfang databases was performed to identify the eligible studies assessing the association between TNF-α rs1800629 polymorphism and risk of cervical lesions. There were no language restrictions and the last search time was the November 16, 2012. We used the keywords and subject terms: (“cervical carcinoma”, “cervical cancer”, “cervix carcinoma” or “cervix cancer” or cervical neoplasm, cervical lesions, cervical lesion, cervical intraepithelial lesion, cervical intraepithelial lesions, cervical dysplasia, cervical intraepithelial neoplasia) and (“polymorphism” or “variant” or “genotype” or “mutation” or) and (“tumor necrosis factor” or “TNF” or “308 G/A” or “TNF-α” or “rs1800629”). All eligible studies were retrieved, and their bibliographies were checked for other relevant publications.

### Inclusion criteria and exclusion criteria

To be eligible for the inclusion criteria in the meta-analysis, the following criteria were used: 1) case-control studies comparing cervical lesions cases with healthy or non-cancer controls; 2) studies evaluating the association between TNF-α rs1800629 polymorphism and risk of cervical lesions; 3) Sufficient genotype data of TNF-α rs1800629 polymorphism were reported. 4) Studies was excluded if they were the following: 1) Case-only studies; 2) Case reports, letters, or reviews; 3) Incomplete data or no usable data were reported; 4) Studies containing overlapping data; 5) Family-based design or related cases and controls were contained.

### Data extraction and quality assessment

The data extraction was performed independently by two reviewers, and the conflicting data extracted were settled by discussion. A standardized form was used in the data extraction from the published studies, and the following data were extracted: first author, year of publication, country, ethnicity, study design, diagnostic criteria, source of cases and controls, number of cases and controls, types of cervical lesions, genotyping methods, frequencies of TNF-α rs1800629 genotypes, and the confirmation of Hardy-Weinberg equilibrium (HWE) in controls. The quality of included studies was assessed by the confirmation of HWE in controls, and studies without the confirmation of HWE in controls were defined as low-quality studies, while studies with the confirmation of HWE in controls were defined as high-quality studies.

### Statistical analysis

Before the effect estimation of associations between the TNF-α rs1800629 polymorphism and risk of cervical lesions, we firstly tested whether the genotype frequencies of TNF-α rs1800629 genotypes in the controls were confirmed with HWE using the χ^2^ test. The strength of the associations between the TNF-α rs1800629 polymorphism and risk of cervical lesions was estimated using odds ratios (ORs) and their 95% confidence interval (95%CIs). The following contrasts for the associations between the TNF-α rs1800629 polymorphism and risk of cervical lesions were evaluated: comparison of the variant allele with ancestral allele (A vs. G); comparison of the variant homozygote with the ancestral homozygote (AA vs. GG); comparison of the variant homozygote combined with the heterozygote vs. ancestral homozygote (AA/GA vs. GG); comparison of the variant homozygote vs. ancestral homozygote combined with the heterozygote (AA vs. GA/GG). The I^2^ statistic to quantify the proportion of the total variation due to heterogeneity were calculated, and a I^2^ value of more than 50% was interpreted as significant heterogeneity among studies [26]. When the effects were assumed to be homogenous, the fixed-effects model was used (Mantel-Haenszel method) [27]. If obvious heterogeneity was present, the random-effects model was used (DerSimonian-Laird method) [28]. Subgroup analysis based on ethnicity was used to explore the possible race-specific effect in the association. Subgroup analyses based on the types of cervical lesions and the quality of included studies were also performed. Potential publication bias was assessed by visual inspection of the funnel plots, in which the standard error of logOR of each study was plotted against its logOR, and an asymmetric plot suggested possible publication bias. In addition, We also performed Egger linear regression test at the P<0.05 level of significance to assess the funnel-plot's asymmetry [29]. All analyses were conducted using STATA (Version 11, StataCorp, College Station, TX). All P-values in the meta-analysis were two-sided, and statistical significance was considered when the P-value was less than 0.05.

## Results

### Study selection and description of included studies

**Figure 1:**
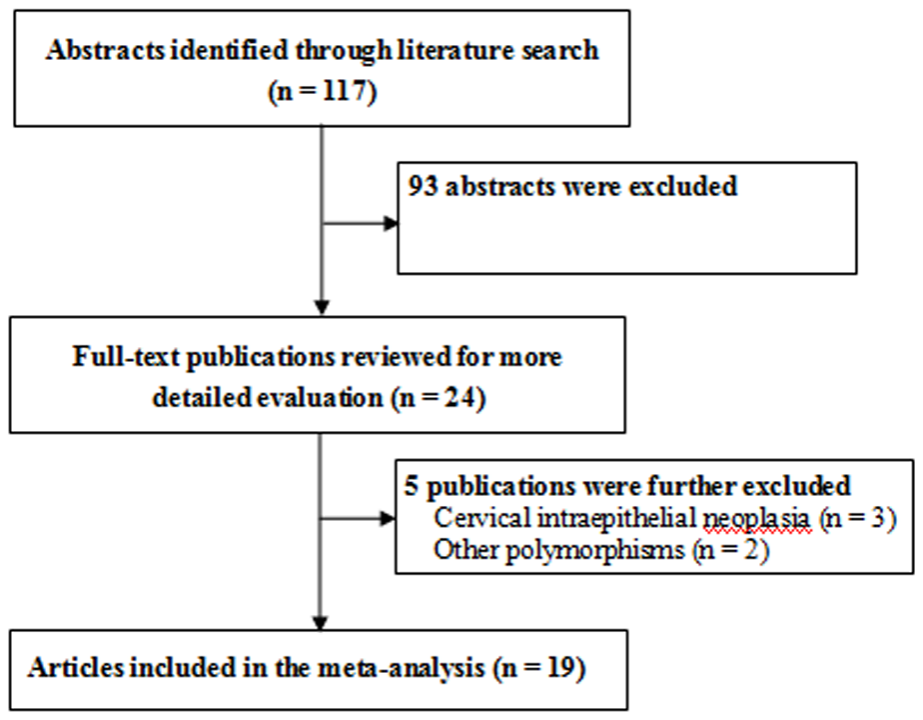
Flow chart of study selection in the meta-analysis of the association between TNF-α rs1800629 polymorphism and cervical lesions risk.

A total of 117 potentially individual abstracts were found from the the Pubmed, Embase, Web of Science, and Wanfang databases, but only 24 studies were preliminarily identified and were further assessed for inclusion [13]–[25], [30]–[39] (Figure 1). After reviewing the full-texts of those 25 studies, three studies were excluded for irrelevant studies [36], [37], [39] and two were excluded for studies assessing the other TNF gene polymorphisms [35], [38]. Since the research by Kohaar *et al.* assessed the associations of TNF-α rs1800629 polymorphism with risks of both cervical cancer and squamous intraepithelial lesions, the data were extracted as two individual studies [21]. Therefore, twenty individual case-control studies from 19 publications with a total of 4,146 cases and 4,731 controls were finally included into the meta-analysis [13]–[25], [30]–[34]. The main characteristics of those twenty studies on the association between TNF-α rs1800629 polymorphism and cervical lesions risk were listed in the Table 1. There were 11 studies from Caucasians, 5 studies from Asians, and 4 ones form mixed populations. Among those 20 studies, most studies 17 studies (85.0%) were about the association between TNF-α rs1800629 polymorphism and invasive cervical cancer, while the left 3 ones were about the association between TNF-α rs1800629 polymorphism and squamous intraepithelial lesions. In addition, 15 from those 20 studies had the confirmation of HWE in controls and were defined as high-quality studies.

**Table 1:** Characteristics of twenty studies on the association between TNF-α rs1800629 polymorphism and cervical lesions risk.

Author, year (Ref.)	Ethnicity	Countries	Type*	Source of controls	Sample size		Genotype method†	Quality
					Case	Control		
Badano I 2012 [12]	Caucasians	Argentina	ICC	Hospital	56	113	PCR-RFLP	High
Barbisan G 2012 [13]	Caucasians	Argentina	ICC	Hospital	122	176	PCR-RFLP	High
Calhoun ES 2002 [14]	Caucasians	USA	ICC	Hospital	127	107	PCR-RFLP	High
Deshpande A 2005 [15]	Caucasians	USA	ICC	Hospital	258	411	PCR-RFLP	High
Duarte I 2005 [30]	Caucasians	Portugal	ICC	Hospital	195	244	PCR-RFLP	High
Fernandes AP 2008 [16]	Mixed	Brazil	SIL	Hospital	42	87	PCR-RFLP	High
Gostout BS 2003 [17]	Caucasians	USA	ICC	Hospital	127	175	PCR-RFLP	High
Govan VA 2006 [18]	Mixed	South Africa	ICC	Hospital	244	228	PCR-RFLP	Low
Huang LL 2012 [31]	Asians	China	ICC	Hospital	42	87	PCR-RFLP	High
Ivansson EL 2010 [19]	Caucasians	Sweden	ICC	Population	1263	804	PCR-RFLP	Low
Jang WH 2001 [20]	Asians	Korea	ICC	Hospital	51	92	PCR-RFLP	High
Kohaar I 2007 [21]	Caucasians	India	ICC	Hospital	120	165	PCR-RFLP	High
Kohaar I 2007 [21]	Caucasians	India	SIL	Hospital	45	165	PCR-RFLP	High
Nieves-Ramirez ME 2011 [32]	Mixed	Mexico	SIL	Hospital	191	205	PCR-RFLP	High
Singh H 2009 [33]	Caucasians	India	ICC	Hospital	150	162	PCR-RFLP	Low
Stanczuk GA 2003 [22]	Africans	Zimbabwe	ICC	Hospital	103	101	PCR-RFLP	High
Wang N 2012 [23]	Asians	China	ICC	Hospital	285	318	PCR-RFLP	Low
Wang Q 2011 [24]	Asians	China	ICC	Hospital	186	200	PCR-RFLP	High
Wang SS 2009 [25]	Caucasians	Costa Rica	ICC	Population	456	800	PCR-RFLP	High
Zu FY 2010 [34]	Asians	China	ICC	Hospital	83	91	PCR-RFLP	Low

(†PCR-RFLP, Polymerase chain reaction restriction fragment length polymorphism; *ICC, Invasive cervical cancer; SIL, squamous intraepithelial lesions.)

### Meta-analysis

**Figure 2:**
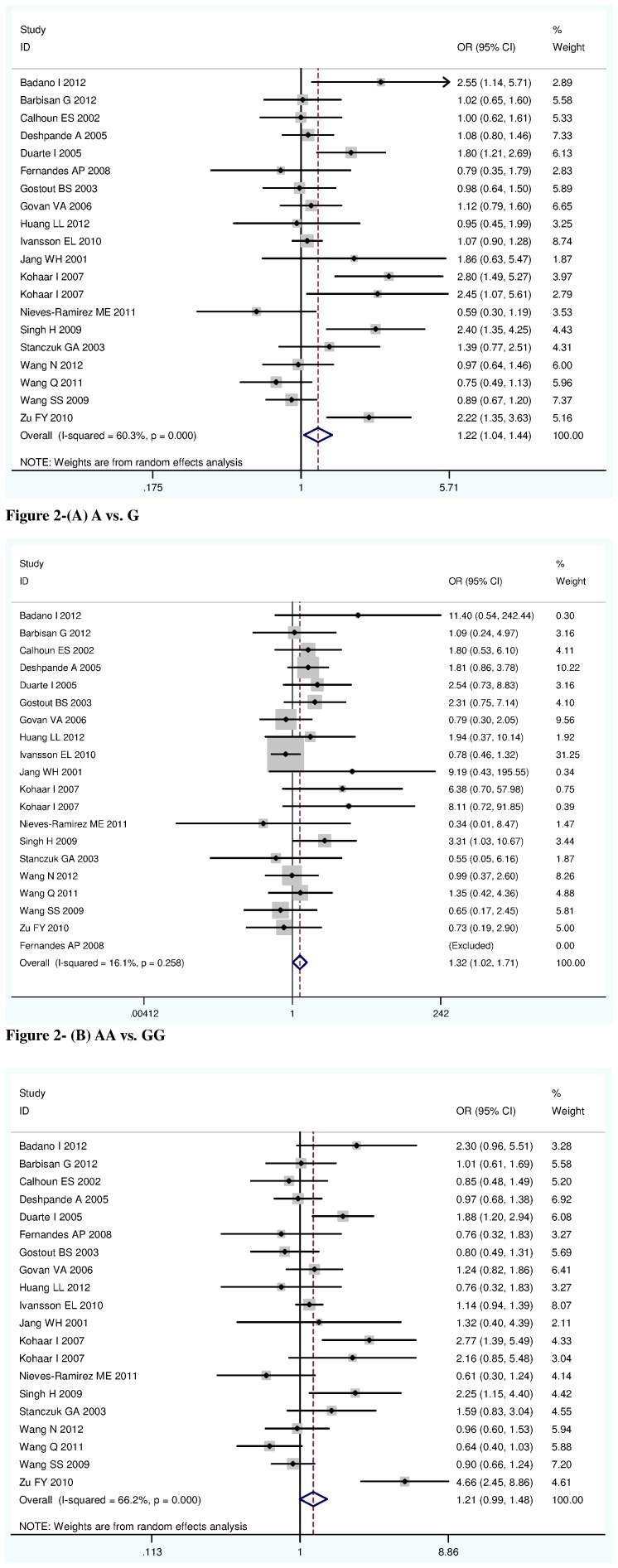
Forest plot describing the association between TNF-α rs1800629 polymorphism and cervical lesions risk. **(Each study is shown by the point estimate of the OR and 95% CI, and (the size of the square is proportional to the weight of each study.)** Figure 2-(A) A vs. G. Figure 2- (B) AA vs. GG. Figure 2- (C) AA/GA vs. GG.

Meta-analysis of all 20 studies showed that there was a significant association between TNF-α rs1800629 polymorphism and cervical lesions risk under two main genetic comparison models (For A versus G: OR 1.22, 95%CI 1.04–1.44, P = 0.017; for AA versus GG: OR 1.32, 95%CI 1.02–1.71, P = 0.034) (Table 2, Figure 2).

**Table 2:** Meta-analysis of the association between TNF-α rs1800629 polymorphism and cervical lesions risk.

Groups	Studies	Subjects (Cases/Controls)	OR [95%CI]	P value	I^2^ value
Total studies					
A vs. G	20	4,146/4,731	1.22[1.04–1.44]	0.017	60.3%
AA vs. GG	20	4,146/4,731	1.32[1.02–1.71]	0.034	16.1%
AG/AA vs. GG	20	4,146/4,731	1.21[0.99–1.48]	0.059	66.2%
AA vs. AG/GG	20	4,146/4,731	1.27[0.98–1.63]	0.071	29.1%
Studies with high quality					
A vs. G	15	2,121/3,128	1.17[0.95–1.45]	0.129	57.8%
AA vs. GG	15	2,121/3,128	1.81[1.26–2.61]	0.001	0.0%
AG/AA vs. GG	15	2,121/3,128	1.09[0.87–1.37]	0.446	55.6%
AA vs. AG/GG	15	2,121/3,128	1.86[1.29–2.68]	0.001	0.0%
Studies with low quality					
A vs. G	5	2,025/1,603	1.35[0.99–1.85]	0.056	72.0%
AA vs. GG	5	2,025/1,603	0.96[0.66–1.39]	0.831	23.1%
AG/AA vs. GG	5	2,025/1,603	1.59[1.02–2.47]	0.040	81.0%
AA vs. AG/GG	5	2,025/1,603	0.86[0.60–1.24]	0.420	42.6%
Caucasians					
A vs. G	11	2,919/3,322	1.33[1.07–1.65]	0.009	67.0%
AA vs. GG	11	2,919/3,322	1.47[1.08–2.00]	0.015	38.0%
AG/AA vs. GG	11	2,919/3,322	1.26[1.01–1.58]	0.045	60.8%
AA vs. AG/GG	11	2,919/3,322	1.45[1.06–1.97]	0.018	39.3%
Asians					
A vs. G	5	647/788	1.17[0.75–1.83]	0.482	68.2%
AA vs. GG	5	647/788	1.24[0.70–2.21]	0.466	0.0%
AG/AA vs. GG	5	647/788	1.23[0.58–2.57]	0.591	84.4%
AA vs. AG/GG	5	647/788	1.08[0.62–1.89]	0.789	30.8%
ICC					
A vs. G	17	3,868/4,274	1.24[1.05–1.47]	0.011	60.4%
AA vs. GG	17	3,868/4,274	1.31[1.01–1.70]	0.043	13.9%
AG/AA vs. GG	17	3,868/4,274	1.25[1.01–1.54]	0.039	68.3%
AA vs. AG/GG	17	3,868/4,274	1.25[0.97–1.62]	0.088	29.4%
SIL					
A vs. G	3	278/457	1.03[0.44–2.39]	0.951	71.6%
AA vs. GG	3	278/457	1.99[0.09–45.32]	0.667	59.2%
AG/AA vs. GG	3	278/457	0.96[0.46–2.02]	0.912	57.7%
AA vs. AG/GG	3	278/457	1.98[0.10–40.61]	0.658	56.4%

(**Abbreviations:** OR, odds ratio; 95%CI, 95% confidence interval; ICC, Invasive cervical cancer; SIL, squamous intraepithelial lesions.)

Subgroup analysis of studies with high quality showed that there was still a significant association between TNF-α rs1800629 polymorphism and increased risk of cervical lesions under two main genetic comparison models (for AA versus GG: OR 1.81, 95%CI 1.26–2.61, P = 0.001; for AA versus GA/GG: OR 1.86, 95%CI 1.29–2.68, P = 0.001) (Table 2). However, in the subgroup analysis of studies with low quality, there was no association between TNF-α rs1800629 polymorphism and increased risk of cervical lesions (Table 2).

Subgroup analysis of Caucasians showed that there was a significant association between TNF-α rs1800629 polymorphism and increased risk of cervical lesions under all four genetic models (For A versus G: OR 1.33, 95%CI 1.07–1.65, P = 0.009; for AA versus GG: OR 1.47, 95%CI 1.08–2.00, P = 0.015; for GA/AA versus GG: OR 1.26, 95%CI 1.01–1.58, P = 0.045; for AA versus GA/GG: OR 1.45, 95%CI 1.06–1.97, P = 0.018). However, subgroup analysis of Asians showed that there was no association between TNF-α rs1800629 polymorphism and increased risk of cervical lesions in Asians (Table 2).

Subgroup analysis by the types of cervical lesions showed that there was a significant association between TNF-α rs1800629 polymorphism and increased risk of cervical cancer (For A versus G: OR 1.24, 95%CI 1.05–1.47, P = 0.011; for AA versus GG: OR 1.31, 95%CI 1.01–1.70, P = 0.043; for AA/GA versus GG: OR 1.25, 95%CI 1.01–1.54, P = 0.039). But there was no association between TNF-α rs1800629 polymorphism and risk of squamous intraepithelial lesions (Table 2).

### Publication bias

**Figure 3:**
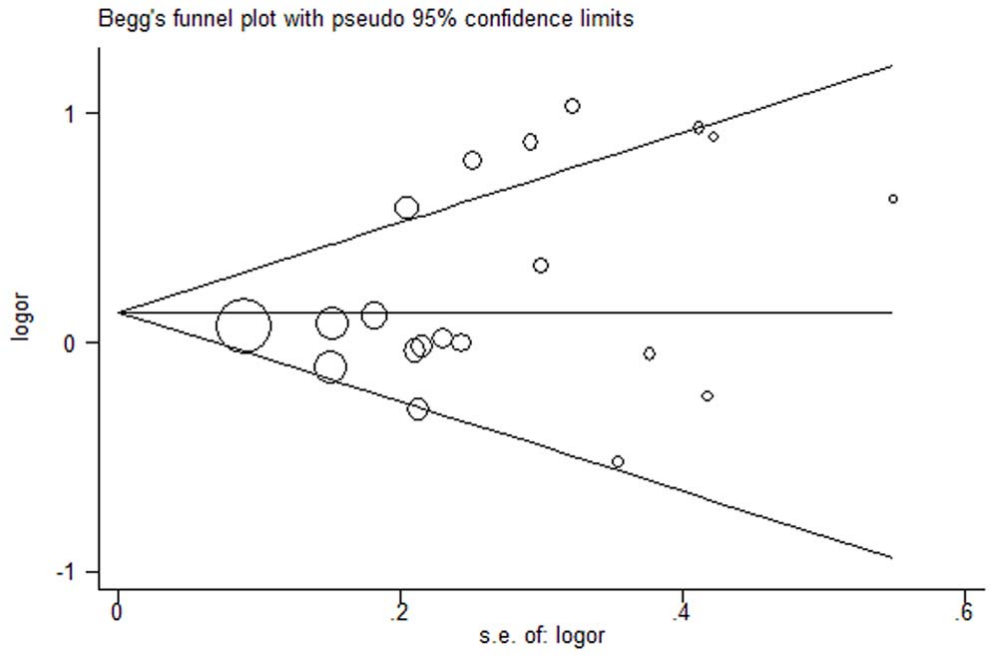
Funnel plot for the detection of the publication bias in this meta-analysis.

Publication bias was investigated by Begg's funnel plot, and funnel plot asymetry was further assessed by Egger's linear regeression test. As shown in the Figure 3, there was low possibility of asymmetry in the allele comparison model (A versus G) of this meta-analysis (Figure 3). Besides, the Egger linear regression test also suggested there was no significant risk of publication bias (P = 0.532). Therefore, there was no risk of publication bias in the meta-analysis.

## Discussion

Cervical cancer is mainly initiated by HPV infection, and TNF-α is an inflammatory cytokine which may play important roles in the progression of cervical lesions [3], [5]. There is a possible association between TNF-α rs1800629 G/A polymorphism and risk of cervical lesions, but previous studies report conflicting results [12]–[25]. We performed a meta-analysis to comprehensively assess the association between TNF-α rs1800629 polymorphism and cervical lesions risk. Twenty case-control studies with a total of 4,146 cases and 4,731 controls were finally included into the meta-analysis [13]–[25], [30]–[34]. Overall, TNF-α rs1800629 polymorphism was significantly associated with increased risk of cervical lesions under two main genetic comparison models (Table 2). Subgroup analysis by ethnicity further showed that there was a significant association between TNF-α rs1800629 polymorphism and increased risk of cervical lesions in Caucasians but not in Asians (Table 2). Subgroup analysis by the types of cervical lesions showed that there was a significant association between TNF-α rs1800629 polymorphism and increased risk of cervical cancer (Table 2). Therefore, the meta-analysis suggests that TNF-α rs1800629 polymorphism is associated with increased risk of cervical lesions, especially in Caucasians.

Cervical cancer is the third most common cancer and the fourth leading cause of cancer death among female in the world. In recent years, the incidence of cervical cancer has increased gradually [2]. Since cervical cancer is considered to be a preventable disease because of its relatively long period of precancerous lesions, identifications of risk factors of cervical lesions can help us find some effective and preventive interventions. It has been well accepted that HPV and smoking are main causes of almost all cases with cervical lesions, and the genetic factors also play important roles in the development of cervical lesions [3]. Currently, several genetic polymorphisms have been suggested to be associated with risk of cervical cancer, such as p53 Arg72Pro mutation and MTHFR gene polymorphisms [40]–[42].

TNF-α is secreted mainly by activated macrophages, which is an extraordinarily pleiotropic cytokine with a central role in immune homeostasis, inflammation, and host defense [5]. The development of cervical cancer is induced by persistent HPV infection, and TNF-α may be involved in the susceptibility to HPV infection and development of cervical cancer by modulating viral replication [5]. TNF-α rs1800629 is the most studied polymorphism, which is a G to A transition in the promoter at position −308 and is associated with the level of TNF-α expression [10], [11]. Although a possible association of the TNF-α rs1800629 polymorphism with risk of cervical lesions was reported, and many case-control studies were further performed to identify the association, it was still unknown whether there was a significant association between TNF-α rs1800629 polymorphism and risk of cervical lesions [13]–[25], [30]–[34]. In current meta-analysis, we conducted a systematic review and meta-analysis of previous published case-control studies to comprehensively evaluate the association between TNF-α rs1800629 polymorphism and cervical lesions risk. The findings from the meta-analysis suggest that TNF-α rs1800629 polymorphism is associated with increased risk of cervical lesions and TNF-α plays an important role in the development of cervical cancer.

There were some limitations in our meta-analysis. Firstly, there was limited number of eligible studies in the meta-analysis of the association between TNF-α rs1800629 polymorphism and risk of squamous intraepithelial lesions. The limited sample size in the meta-analysis may fail to provide enough statistical power to detect a possible or weak effect of TNF-α rs1800629 polymorphism on squamous intraepithelial lesions. Therefore, more studies with large sample are needed to give a more precise estimation of the association between TNF-α rs1800629 polymorphism and risk of squamous intraepithelial lesions. Secondly, the meta-analysis was based on unadjusted data, as the ORs adjusted for the main confounding variables were not available from those studies. To provide a more reliable estimation of the association, more studies with well-design and large sample size are needed to further identify the association. Finally, gene-gene interactions were not fully addressed in the meta-analysis for the lack of relevant data. There are several polymorphisms associated with risk of cervical cancer, such as p53 Arg72Pro mutation and MTHFR gene polymorphisms [40]–[42]. But no studies investigated the gene-gene interactions in the association between TNF-α rs1800629 polymorphism and risk of cervical lesions. Future studies may further assess the possible gene-gene interactions in the association between TNF-α rs1800629 polymorphism and risk of cervical lesions.

In summary, our meta-analysis suggests that TNF-α rs1800629 polymorphism is associated with increased risk of cervical lesions, especially in Caucasians. However, more studies with large sample and well-design are needed to give a more reliable estimation of the association between TNF-α rs1800629 polymorphism is associated and risk of cervical lesions.

## Supporting Information

Checklist S1 **PRISMA 2009 checklist.**
(DOC)
